# Neuronal activity-regulated gene transcription: how are distant synaptic signals conveyed to the nucleus?

**DOI:** 10.12688/f1000research.1-69.v1

**Published:** 2012-12-19

**Authors:** Miriam Matamales

**Affiliations:** 1 Centre for Ageing Dementia Research, Queensland Brain Institute, The University of Queensland, St Lucia, QLD 4072, Australia

## Abstract

Synaptic activity can trigger gene expression programs that are required for the stable change of neuronal properties, a process that is essential for learning and memory. Currently, it is still unclear how the stimulation of dendritic synapses can be coupled to transcription in the nucleus in a timely way given that large distances can separate these two cellular compartments. Although several mechanisms have been proposed to explain long distance communication between synapses and the nucleus, the possible co-existence of these models and their relevance in physiological conditions remain elusive. One model suggests that synaptic activation triggers the translocation to the nucleus of certain transcription regulators localised at postsynaptic sites that function as synapto-nuclear messengers. Alternatively, it has been hypothesised that synaptic activity initiates propagating regenerative intracellular calcium waves that spread through dendrites into the nucleus where nuclear transcription machinery is thereby regulated. It has also been postulated that membrane depolarisation of voltage-gated calcium channels on the somatic membrane is sufficient to increase intracellular calcium concentration and activate transcription without the need for transported signals from distant synapses. Here I provide a critical overview of the suggested mechanisms for coupling synaptic stimulation to transcription, the underlying assumptions behind them and their plausible physiological significance.

## Introduction

Among the hundreds of distinct cell types that make up our bodies, neurons are the most morphologically complex, and also one of the most dynamic in their responsiveness and adaptability. Neurons contain structural specialisations, which allow the rapid processing and transmission of the thousands of synaptic inputs that they simultaneously receive. The vast majority of excitatory synapses involving the neurotransmitter glutamate are made into dendrites, which are extensions of the plasma membrane that can span hundreds of microns and cover broad fields in the tissue ^1^. Release of glutamate into the synaptic cleft induces transient postsynaptic electrical and biochemical responses, which can eventually promote stable changes in the properties of the neuron ^2^. At an excitatory synapse, glutamate acts on both metabotropic (mGluRs) and the ionotropic N-methyl-D-aspartate (NMDA) and α-amino-3-hydroxy-5-methyl-4-isoxazolepropionic acid (AMPA) receptors ^3, 4^. mGluRs are G-protein coupled receptors (GPCRs) that are linked to heterotrimeric G proteins on the intracellular side of the membrane. Activation of mGluRs by glutamate modulates the activity of various signal transduction pathways through the change in concentration of intracellular second messengers such as intracellular inositol 1,4,5-trisphosphate (IP _3_) and cyclic adenosine monophosphate (cAMP), which controls the cAMP-dependent protein kinase (PKA) ^5^. Glutamate binding to AMPA receptors (AMPARs) opens the ion channel and induces fast depolarisation of the postsynaptic membrane, mainly through the influx of sodium (Na ^+^) ions. By contrast, glutamate-gated channel opening of NMDA receptors enables calcium (Ca ^2+^) influx into the dendritic spine, which initiates a cascade of signalling events involving the stimulation of the Ca ^2+^/calmodulin-dependent protein kinase (CaMK) as well as the extracellular signal regulated kinase (ERK). The stimulation of CaMK and ERK triggers the phosphorylation-induced activation of a myriad of cellular targets including ion channels and transmembrane receptors, which in turn modifies their conductance properties ^6, 7^.

Additionally, an important consequence of the activation of these signalling pathways upon excitatory neurotransmission is that they can regulate the activity of nuclear factors thereby triggering changes in gene transcription ^8^. The mechanism by which neuronal activity is conveyed to the nucleus for the induction of activity-dependent gene expression programs is one of the most investigated topics in neuroscience, since it is believed to be necessary for the establishment of memories. Indeed, mRNA synthesis inhibitors such as actinomycin D have been shown to prevent late long-term potentiation in hippocampal slices and to impair retention of new memories in several species and learning paradigms ^9– 13^. Interestingly, former studies regarding activity-dependent regulation of transcription showed that induction of genes occurs within a few minutes after excitatory electrical and pharmacological stimulation ^14– 17^. These studies demonstrate that, in spite of the highly polarised morphology of neurons, synaptic signals are rapidly conveyed to the nucleus to allow the immediate regulation of gene transcription.

Various mechanisms have been proposed that might couple synaptic activity to gene transcription ^18– 20^. The main difference among these models relies on the nature of the signal that carries the message, i.e. synapto-nuclear messengers, IP _3_-triggered Ca ^2+^ waves or action potentials. This review will critically discuss the primary experimental findings supporting the current models for communication between synapses and the nucleus and ascertain their potential role in efficiently and timely coupling neuronal activity to gene expression.

## Translocation of transcription regulators from synapses to the nucleus

The restriction of effector proteins to particular subcellular locations is a commonly employed strategy used by most known signal transduction cascades, and allows coordination of signalling events in space and time ^21– 23^. In many cases, the activation of signalling proteins by upstream regulators, or their activation of downstream effectors, involves their rapid and regulated translocation to specific subcellular compartments ^24^. Indeed, nuclear translocation of signalling molecules is known to play a role in the timely expression of genes in response to extracellular stimuli ^25– 27^. For example, although PKA and ERK are preferentially distributed in the cytoplasm in resting conditions, the rise of intracellular second messengers drives their rapid accumulation in the nucleus, where they phosphorylate multiple nuclear targets ^28, 29^. Furthermore, transcription factors can also relocate from the cytoplasm to the nucleus in response to stimuli that induce apoptosis, cell differentiation or proliferation ^30– 32^. There are several advantages of moving signalling proteins between neighbouring subcellular compartments upon cellular activity, including the enhancement of the specificity as well as speed of signal transmission ^22– 34^. Interestingly, there is strong evidence demonstrating that a variety of stimuli mimicking neuronal activity, such as bath application of neurotransmitters or electrical stimulation, also induce the cyto-nuclear translocation of signalling proteins in primary neuronal cultures and brain slice preparations ^35, 36^. Moreover, the robust accumulation of gene transcription regulators in the nucleus is also observed in response to a wide variety of physiological and pharmacological stimuli *in vivo*. For example, immunohistological analysis of mouse brain sections have shown that the rapid nuclear accumulation of activated ERK in various regions of the brain including the hippocampus, amygdala and projection neurons of the striatum, occurs in animals that have been trained to behavioural paradigms that underlie learning, or following acute administration of addictive and non-addictive drugs ^37– 41^.

## Synapse-to-nucleus protein translocation

**Figure 1.**
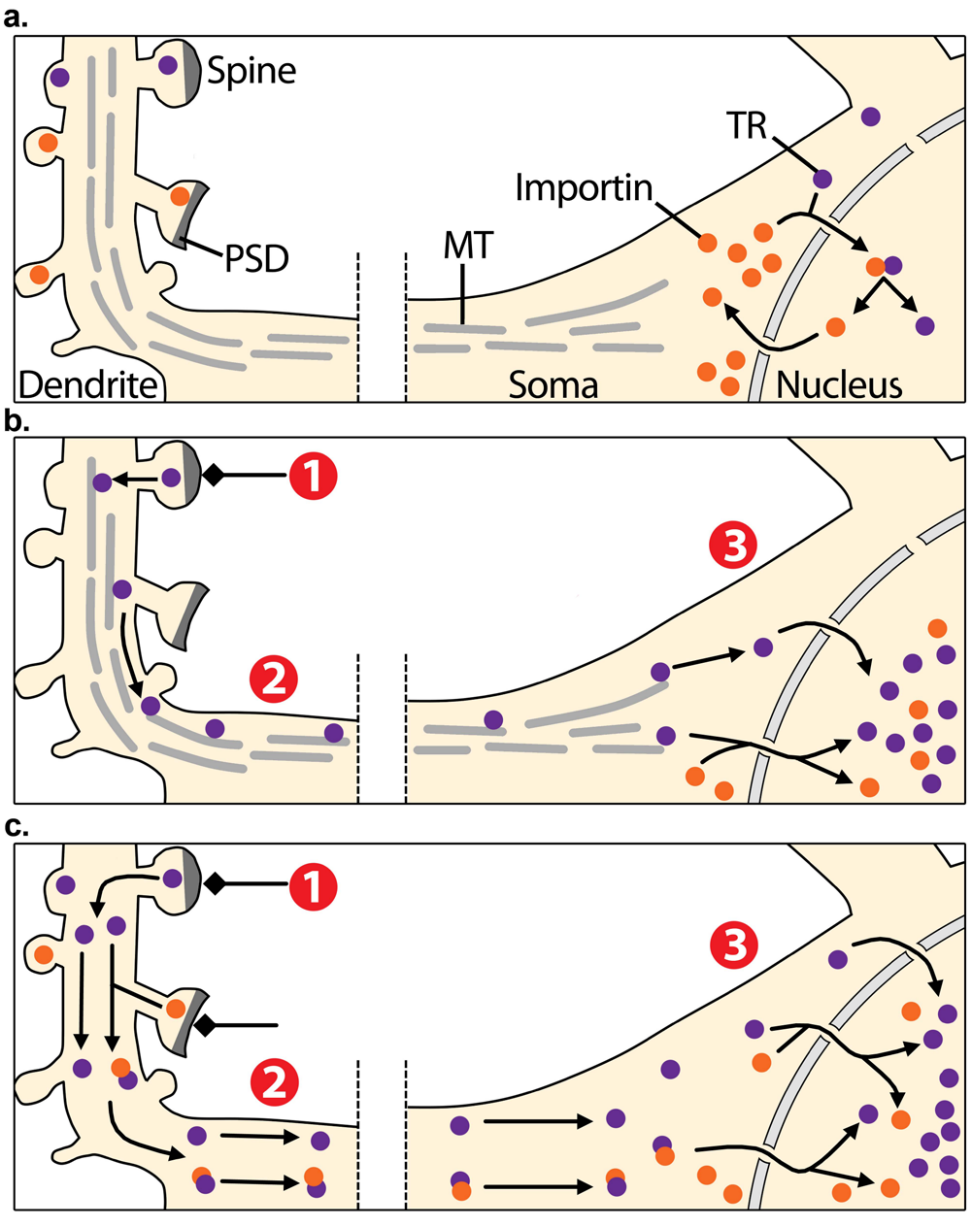
Synapto-nuclear translocation of transcription regulators. **a**) In non-stimulated conditions, transcription regulators (TR, purple dots) are localised into distant dendrites as well as at the perinuclear zone. Some TRs are transported from the cytoplasm to the nucleus by importins (orange dots). Importins are also distributed across distant dendrites and at the postsynaptic density (PSD). **b, c**) Excitatory inputs that stimulate synapses (1) are believed to activate TRs, which are then transported to the soma along the neuron (2) through microtubule-based active transport **b**) or by passive diffusion **c**). Excitatory inputs are also proposed to activate importins at the PSD, which then associate to synaptic cargoes and facilitate their synapto-nuclear translocation **c**). Therefore, this model predicts that synaptic activity triggers the accumulation of TRs from distant synapses in the nucleus, promoting the induction of gene expression programs (3).

Although the translocation of proteins has been demonstrated to occur between adjacent subcellular compartments i.e. from the cytoplasm to the plasma membrane or from the cytoplasm to the nucleus, it has been hypothesised that it can also occur between more distant compartments, linking dendritic terminals and axon tips with the nucleus ^42, 43^. Indeed, the retrograde transport of signals from distal axons to neuronal cell bodies by motor-dependent transport along microtubules is one of the best known mechanisms for long-distance communication ^44^. So far, these mechanisms have been studied in the context of development, survival and axonal injury ^45^. However, recent studies have proposed that activity at excitatory synapses can also promote the movement of proteins from distant parts of the dendritic arbour to the nucleus (Reviewed in ^18, 20, 46^). This idea of long distance rather than local signalling in response to synaptic inputs provides a tantalising model in which the transport of transcription regulators from stimulated synapses serves to inform the genome about peripheral neuronal activity (Figure 1).

Former experimental evidence supporting this hypothesis comes from early studies of brain cell fractionation that were published by different laboratories in the 1990s ^43, 47, 48^. Intriguingly, it was reported that the nuclear factor κ light chain enhancer of activated B-cells (NF-κB) can be detected in the synaptosomal fraction of cortical and hippocampal preparations, which contains pre- and post-synaptic elements of neurons. Additional subsequent studies have later shown that other transcription factors are also located at synaptosome preparations, including the cAMP response element-binding protein (CREB) ^49^, CREB2 ^50^ and CREB-regulated transcription coactivator 1 (CRTC1) ^51^. Additionally, fluorescence microscopy analyses of endogenous proteins or fluorescently tagged chimeric constructs conducted to determine the subcellular localisation of these transcription regulators in neurons indicate that they are indeed not confined to the vicinity of the nucleus, but rather extend throughout the whole cytoplasm, reaching distant compartments like dendritic spines. Provided that the only recognised function of these transcription factors takes place in the nucleus where their known cellular targets belong, the intriguing question of how they can be efficiently and timely shuttled to the nucleus to influence transcription arises naturally.

Martin and colleagues have proposed a mechanism for the shipping of activated transcription regulators between synapses and the nucleus ^52, 53^. It involves the participation of members of the karyopherin family of nuclear transport receptors, which are soluble carriers that mediate the regulated translocation of proteins across the nuclear envelope ^54, 55^. The active import of proteins from the cytoplasm to the nucleus is mostly driven by a heterodimeric carrier composed of importin-β and its adaptor importin-α. Importins discriminate their cargo from other cellular proteins by recognition of amino acid targeting sequences known as nuclear localisation signals (NLSs), which contain one or two clusters of basic residues. Monopartite NLSs, exemplified by the SV40 large-T antigen ^56^, have a single cluster of 4–5 basic residues (e.g. P *KKKRK*V), whereas bipartite NLSs, such as that of nucleoplasmin ^57^, have a second basic cluster located 10–12 residues downstream from the first cluster (e.g. *KR*PAATKKAGOA *KKK*LD). Interestingly, immunofluorescence studies have detected importins far away from the nucleus, including dendrites and the axon of mouse hippocampal and *Aplysia* sensory neurons, suggesting a role in long-distance trafficking of proteins towards the nucleus ^52^. Furthermore, strong accumulation of importins in the nucleus is observed in response to stimuli that mimic synaptic activity in neuronal primary cultures and hippocampal slice preparations. These observations led to the hypothesis that importins may direct the translocation of synaptic proteins to the nucleus ^53^. In this line, a study by Holmes and co-workers reported that the cytoplasmic tail of the NR1-1a subunit of the NDMAR contains a putative bipartite NLS which is surrounded by four phosphorylation sites i.e. PDP *KKK*ATFRAITSTLASSF *KRRR*SSKDT ^58, 59^. Based on these results, subsequent co-immunoprecipitation experiments by Jeffrey and colleagues showed that importin-α binds to NR1 *in vitro*, and that this interaction is disrupted by the activity-dependent phosphorylation of the residues flanking the NLS of NR1-1a ^60^. The authors presented a model in which importins are normally tethered to the NLS of the NMDAR in non-stimulated synapses, placing them in close proximity to synaptically localised transcription regulators. Upon stimulation, phosphorylation of the NR1-1a tail by PKA and protein kinase C (PKC) promotes the release of bound importins that are then free to bind soluble synaptic NLS-containing cargoes such as the transcription factor CREB2 ^50^. In view of this appealing mechanism for the long-range transport of proteins to the nucleus, a series of proteomic and bioinformatic studies have been conducted in order to identify novel NLS-containing proteins undergoing importin-mediated synapto-nuclear translocation ^61^. Surprisingly, around 166 out of 1100 proteins from postsynaptic density (PSD) purified fractions were predicted to contain putative NLSs ^62^. Among them, there were found several proteins involved in RNA trafficking and splicing, as well as regulators of transcription factor activity ^61, 63^.

## Limitations and challenges to the model

Although a number of arguments support the existence of synapto-nuclear protein messengers, there are many remaining concerns that challenge the synapto-nuclear translocation model. One important caveat to be considered regards the identification of potential synapto-nuclear messengers by mass spectrometry. It has been observed that due to the high sensitivity of protein identification by this method, low-level contaminants introduced during the biochemical purification of brain fractions are commonly detected ^64^. Moreover, searching for synapto-nuclear messengers by NLS identification may yield false positive results ^61^, since similar clusters of basic residues are present in kinase and phosphatase docking sites and help to mediate protein-protein interaction ^65^. For example, the consensus phosphorylation site for PKC is R/K _1–3_, X _2–0_-S/T whereas for PKA, it is R-R-X-S/T-Φ, where X is any amino acid and Φ is a hydrophobic residue which closely resembles the above mentioned NLS found in the NR1-1a subunit of the NMDA receptor ^66^. Accordingly, a detailed functional dissection of these motifs needs to be performed before concluding that they represent *bona fide* NLSs. Experiments tracking fluorescently tagged proteins through live-cell imaging techniques as well as the use of nuclear import assays in permeabilised cells should be routinely used in order to confirm the possible nuclear translocation of these proteins ^67– 69^.

Another point that needs to be directly demonstrated is whether transcription regulators distributed at distant parts of the neuron actually shuttle to the nucleus. Recent experiments using cultured hippocampal neurons that express a photoconvertible fluorescent form of the synapto-nuclear transcription factor CRTC1 indeed suggest that activation of distant dendrites by local glutamate uncaging can promote its accumulation to the nucleus ^51^. However, the movement of single synaptic messengers on route to the nucleus has never been directly tracked, and therefore this point remains to be clarified. The combination of super-resolution fluorescence microscopy with live-cell single-molecule-based imaging may provide new insights into the dynamics of the long-range transport of proteins in neurons ^70^.

An important question regarding the long-range transport of proteins to the nucleus concerns the speed at which these messengers may travel towards the soma, which is crucial to explain their potential involvement in timely coupling of neuronal activation to the robust induction of gene programs ^71^. It has been estimated that a transcription factor involved in the rapid induction of activity-dependent genes should travel at nearly 75 microns per minute towards the nucleus, but NF-κB has been calculated to move at only 2 microns per minute ^72^. It is currently unknown whether such a rapid movement of proteins from synapses to the nucleus may occur by diffusion, facilitated transport, signalling endosomes or whether it requires association with the microtubule network. Moreover, a significant limitation of long-range signalling by the synapto-nuclear shuttling of transcription factors is that the signal is not amplified nor regenerated on its way towards the nucleus. Distantly stimulated messengers are likely to be inactivated or lost in transit, thereby compromising the direct involvement of synaptic activity on the control of nuclear functions. For example, if a transcription factor is activated by phosphorylation at the synapse, protein phosphatase activity will likely cause its inactivation on its way to the nucleus. Finally, a critical issue that needs to be addressed is to determine the advantage, if any, of mobilising transcription regulators from distant synapses over those already located adjacent to the nucleus to control gene expression.

Despite the list of synapto-nuclear messengers is rapidly growing, there are considerable ambiguities in the synapse-to-nucleus translocation model that need to be clarified. Further research is essential to gain a more accurate understanding of the presumed role of these signalling mechanisms on the coupling of excitatory synaptic transmission to gene induction during neuronal plasticity.

## Propagation of calcium waves from synapses to the nucleus

It is now well documented that the rise of Ca ^2+^ concentration in the nucleus represents an essential step for the activity-dependent induction of gene expression programs ^19, 73– 75^. Cytoplasmic Ca ^2+^ concentration can increase via voltage- and ligand-gated channels as well as by release from intracellular Ca ^2+^ stores ^76^. The main internal Ca ^2+^ store is the endoplasmic reticulum (ER), which acts both as a sink for Ca ^2+^ that enters from the extracellular space, and as a source for Ca ^2+^ release into the cytosol. The ER forms a continuous vesiculo-tubular system that constitutes an elaborated network distributed throughout the cytoplasm ^77^. In neurons, this network extends from the outer nuclear envelope in the soma into axonal processes and dendritic arborisations, including spines ^78, 79^. Release of Ca ^2+^ from the ER can occur upon increase of intracellular IP _3_ and adenosine diphosphate ribose (ADP-ribose), which bind to and open IP _3_ receptors (IP _3_Rs) and ryanodine-type receptors (RyRs), respectively ^80^. As mentioned above, IP _3_ can be generated by the stimulation of mGluRs which can be coupled to phospholipase C (PLC), the enzyme that cleaves the membrane phospholipid phosphatidylinositol 4,5-bisphosphate to form IP _3_^81, 82^. Moreover, Ca ^2+^ itself activates the opening of RyRs and enhances the Ca ^2+^-releasing action of IP _3_, therefore stimulating the efflux of more Ca ^2+^ from the ER lumen ^83^. This process of Ca ^2+^-induced Ca ^2+^ release (CICR) is believed to establish regenerative Ca ^2+^ waves that spread bidirectionally from their initiation site and was first discovered in skeletal muscle ^84, 85^. Indeed, CICR is considered to be the physiological mechanism of Ca ^2+^ release in cardiac muscle, playing a major role in cardiac excitation-contraction coupling ^86^.

The development of fluorescent Ca ^2+^ indicator molecules, photolysis techniques and laser-scanning microscopy has enabled the investigation of the Ca ^2+^ dynamics in neurons at very fine temporal and spatial resolutions ^87– 90^. Synaptically activated, IP _3_-mediated propagation of Ca ^2+^ release has been observed in pyramidal neurons in the rodent hippocampus (CA1 and CA3 regions), medial prefrontal cortex and principal neurons in the amygdala ^91– 95^. Depending on the neuronal cell type analysed and the stimulation protocol used, dendritic Ca ^2+^ waves can be highly localised or can spread to a larger extent ^96^. For example, when IP _3_ is generated by focal synaptic stimulation with metabotropic agonists, the generated response is weak and restrained ^97– 99^. By contrast, when IP _3_ production is stimulated by bath application of such agonists, a higher response can be evoked and a rise in Ca ^2+^ concentration can be observed in the nucleus ^99, 100^.

**Figure 2.**
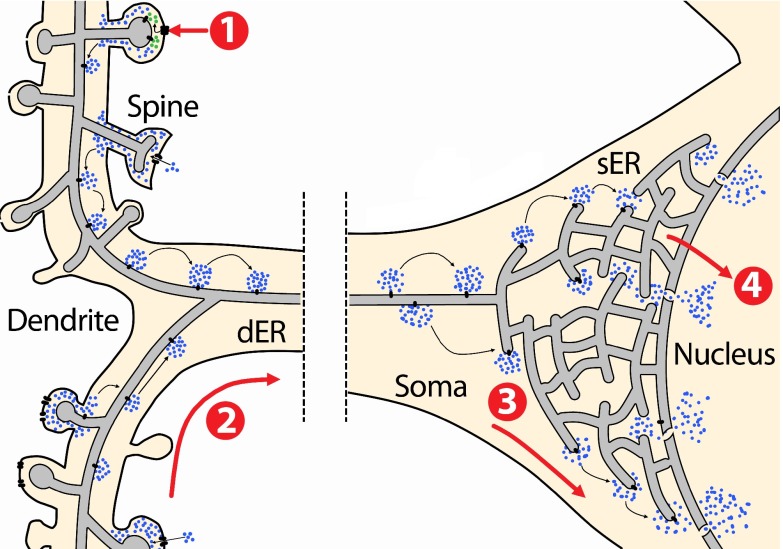
Calcium signal propagation from the synapse to the nucleus. The endoplasmic reticulum (ER) is distributed throughout the cytoplasm from the nuclear envelope to dendritic spines. Excitatory synaptic stimulation through glutamate causes membrane depolarisation and entry of Ca ^2+^ (blue dots) from the extracellular space through NMDARs (1). Moreover, glutamate also activates mGluRs coupled to PLC thereby stimulating IP _3_ production (green dots). Ca ^2+^ and IP _3_ stimulate receptors present at the ER membrane that open and release more Ca ^2+^ from the ER lumen, establishing a Ca ^2+^-induced Ca ^2+^-release wave (2) that propagates from dendrites towards the soma. In the soma, Ca ^2+^ release from the ER activates cytoplasmic Ca ^2+^-dependent signalling cascades that convey the signal to the nucleus (3). Moreover, Ca ^2+^ can be released to the nucleus from the ER, where it activates nuclear transcription regulators (4).

These observations have led to the hypothesis that excitatory inputs trigger IP _3_R-dependent CICR waves that are able to spread forward from the distant synapses towards the soma and mediate the release of Ca ^2+^ from the inner membrane of the nuclear envelope into the nucleus (Figure 2) ^76^. Therefore, it has been suggested that the process of CICR may serve to communicate between distant cellular compartments in neurons and serve as a means to mediate the coupling of synaptic activity to gene transcription. While this model has the advantage over the synapto-nuclear translocation model of being a regenerative process in which the synaptic signal does not decay over great distances, evidence for the distant communication by this mechanism is controversial. First, it is not well established whether functional Ca ^2+^ channels are present in the nuclear envelope in neurons, and if they can indeed release Ca ^2+^ into the nucleoplasm ^101– 103^. More detailed studies on the spatial distribution of receptors involved in Ca ^2+^ release from the ER in neurons may resolve such discrepancies. Second, it is important to note that whereas Ca ^2+^ alone is sufficient to cause its own release through RyRs ^104– 106^, it cannot do so through IP _3_Rs. In the case of IP _3_Rs, Ca ^2+^ can cause self-release only in the presence of IP _3_, thus the availability of IP _3_ in the cytoplasm is a major factor determining the extent of Ca ^2+^ wave propagation ^107– 109^. Because of these findings, CICR is generally considered as an exclusive property of RyRs, but not of IP _3_Rs, even though IP _3_R exhibits the apparent CICR behavior in the presence of IP _3_^86, 110, 111^.

As noted above, experiments in different types of neurons suggest that the spread of IP _3_-mediated Ca ^2+^ waves depends on the availability of IP _3_ in the preparation ^96^. Although IP _3_ has a fast diffusion constant, it has a limited spatial range of action as it is rapidly metabolised by 3-kinase and 5'-phosphomonoesterase and it is estimated to have a lifetime of a few seconds ^112, 113^. Moreover, during the past few years it has become clear that there are functional compartments existing within dendritic trees that control the diffusion of intracellular messengers, particularly within spines ^87, 114^. Hence, the restriction of mobilised IP _3_ to sites close to activated synapses makes it unlikely that synaptically triggered IP _3_-dependent CICRs spread to the soma and transport a message from the synapse. However, functional metabotropic receptors coupled to IP _3_ production are present at the level of the soma in the plasma and the inner nuclear membranes of several neuron types, raising the possibility that local rather than remotely generated IP _3_-dependent release of Ca ^2+^ from the ER may contribute to the induction of gene transcription ^115– 118^.

## Propagation of electrical signals along the plasma membrane

The models discussed in the previous sections are built on the assumption that activation of postsynaptic glutamate receptors triggers the transmission of intracellular messengers (i.e. transcription regulators and Ca ^2+^ waves) from distant dendritic sites to the nucleus. However, several lines of evidence have suggested that gene expression can be triggered by NMDAR and mGluR-independent mechanisms ^119, 120^. Experiments using dorsal root ganglion cell culture preparations, which are devoid of synapses, demonstrated that phosphorylation of nuclear factors and induction of genes could be induced with electrical stimulation ^121^. Furthermore, electrophysiological studies using hippocampal slice preparations have shown that somatic action potentials can trigger gene transcription without the involvement of synaptic activation and in the presence of NMDAR antagonists ^119, 120, 122^. However, NMDARs appeared to have a role in the activation of nuclear events through their contribution to action potential generation ^122^. These results led to a third model proposed by Dudek and colleagues, which posits that the robust depolarisation of the somatic plasma membrane is sufficient to induce gene transcription without the involvement of transported biochemical signals from distant synapses ^71, 72, 123^. According to this model, transmission of synaptic events from distant compartments to the soma is achieved through the propagation of electrical signals over long distances across the plasma membrane. There are several mechanisms of long-range electrical propagation that regulate the degree of depolarisation at the soma. A simplified explanation of the process is briefly provided here, however the reader is referred to the excellent available reports fully addressing this topic ^124, 125^. On one hand, excitatory synaptic events lead to changes in the membrane conductance that can be forward-propagated to the soma in the form of dendritic spikes ^126, 127^. On the other hand, action potentials initiated at the axon initial segment can be retrogradely propagated into the soma and the dendritic tree, spreading back the electrical signal ^128^. The range of propagation efficacies of dendritic spikes and backpropagated action potentials varies in different cell types and is influenced by multiple passive and active properties of the somatodendritic compartment, including dendritic geometry, voltage-gated channel densities and the spatial and temporal profile of synaptic excitation and inhibition ^125, 129, 130^.

It is well established that membrane depolarisation in the soma causes the opening of voltage-gated Ca ^2+^ channels (VGCCs), which allow the influx of Ca ^2+^ from the extracellular space to the cytoplasm, thus coupling synaptic activity to intracellular signalling ^14, 131^. Interestingly, it appears that, among the different classes of VGCCs, L-type VGCCs seem to be significantly involved in regulating transcription since activity-dependent induction of genes is supressed by exposure to specific antagonists and increased by (-)BayK-8644, a VGCC agonist ^14, 132, 133^. The differential ability of L-type VGCCs to preferentially convey signals to the nucleus over the other types of VGCCs may be due to their relatively slow inactivation rate, high single-channel conductance for Ca ^2+^ and relative localisation to Ca ^2+^ intracellular sinks ^134, 135^. In addition, Ca ^2+^ entry through L-type VGCCs has been suggested to trigger ER-directed Ca ^2+^ waves that propagate from the plasma membrane of the soma to the nucleus without the involvement of cyto-nuclear translocation of proteins ^73, 76^. These findings suggest that L-type VGCCs at the soma are well suited for coupling synaptic excitation to activation of transcriptional events.

**Figure 3.**
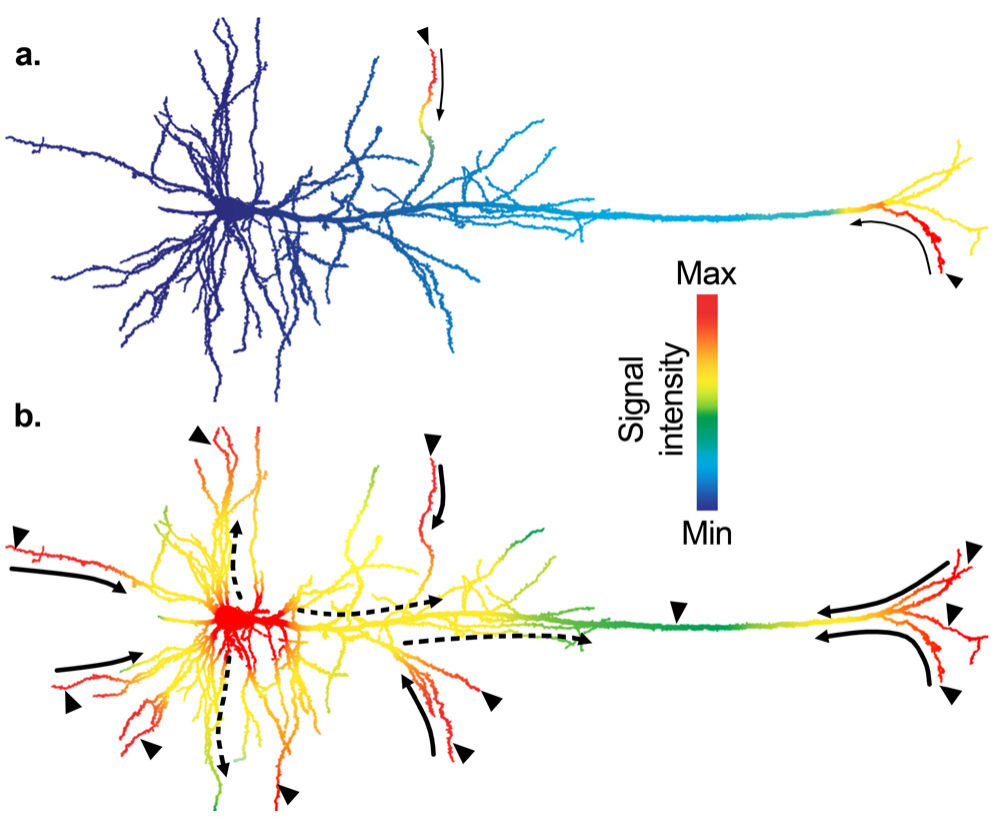
Activation of nuclear functions by action potentials. Excitatory synaptic activity (arrowheads) generates local changes in membrane conductance, which causes the opening of voltage gated calcium channels (VGCCs). A rise in intracellular Ca ^2+^ triggers the local activation of Ca ^2+^-dependent signalling pathways thereby, coupling membrane depolarisation to intracellular signalling. **a**) When neurons receive weak excitatory inputs (small arrowheads), the signal spread is small (thin solid arrows) and the threshold to trigger action potentials is not reached, thus somatic L-VGCCs remain closed. **b**) When neurons receive strong synaptic inputs (big arrowheads), dendritic spikes can efficiently spread (thick solid arrows) in the forward direction and facilitate the initiation of action potentials at the axonal initial segment. Action potentials are backpropagated to the soma and dendrites (dashed arrows), locally generate intracellular Ca ^2+^-dependent signalling cascades. Ca ^2+^ entry in the soma through L-VGCCs will promote the activation of protein effectors that regulate the transcription of plasticity-related genes. In this diagram, the local signal intensity representing both the electrical activity of the membrane and its coupled intracellular signalling is coded by colour. Drawings are adapted from a reconstructed biocytin-filled layer V neuron in the rat cortex (courtesy of B. Chieng and J. Bertran-Gonzalez).

It is important to note that the degree of membrane depolarisation, and thus the opening of L-type VGCCs, is influenced by the immediate electrical history of the neuron. Because postsynaptic events to individual synaptic inputs are usually small and transient, action potential initiation and dendritic spikes require strong synchronous synaptic activation to be generated ^124^. Moreover, inhibitory inputs also affect the way in which excitatory synaptic inputs summate in space and time. Thus, the elaborate integration of excitatory and inhibitory inputs will govern the opening of VGCCs, thereby shaping the activation of Ca ^2+^-dependent nuclear functions ^125^. Taken together, the model upheld by Dudek and colleagues provides a cellular mechanism by which synaptic activity is coupled to gene transcription through VGCCs, which transduce electrical signals into biochemical pathways (Figure 3). Through this mechanism, signalling molecules are only required to translocate between neighbouring subcellular compartments, as observed in many other cell types, conveying information timely and efficiently.

Importantly, this model predicts that action potentials will fail to induce gene transcription programs unless firing above a critical threshold, suggesting that intracellular mechanisms must exist to assess the degree of synaptic stimulation ^123^. Knowledge of how the nucleus computes action potentials to promote gene transcription while avoiding background stimulation is therefore fundamental for understanding how synaptic inputs regulate activity-dependent gene transcription and neuronal plasticity.

## Conclusion

Although it has been known for more than 25 years that gene induction upon excitatory transmission is a required step for neuronal plasticity, it is not yet clear whether a direct link between local synaptic activation and gene transcription exists. An increasing body of literature suggests that there are at least three different pathways by which neurons may inform the nucleus about the events happening at distant dendritic compartments ^18– 20^. In addition to the unique feature of neurons of communicating messages over long distances by electrical signals, it has been recently proposed that the long-range physical translocation of proteins from remote dendritic sites towards the soma can convey information to the nucleus ^20, 46^. The current evidence for the synapto-nuclear translocation model shows that transcription regulators, which were previously considered to be largely localised in close proximity to the nucleus, may also be present at remote sites from the nucleus, in postsynaptic dendritic compartments. Moreover, different stimuli that promote neuronal excitation trigger the accumulation of these gene transcription regulators in the nucleus, although it is still unclear whether they are the same ones activated at distant parts of the neuron. Several critical questions remain enigmatic, as to how and how fast the synapto-nuclear messengers are shipped to the nucleus, and what their actual roles are on gene induction. Taken together, and given the experimental data now available, it is very unlikely that this mechanism participates significantly in the activity-dependent regulation of gene transcription. Future experimental evidence would help to clarify their involvement in other aspects of neuronal physiology.

The most plausible scenario for relaying synaptic activity to the nucleus involves the robust propagation of action potentials, which are initiated upon integration of excitatory, inhibitory and modulatory inputs ^123^. Influx of Ca ^2+^ from the extracellular space through ligand-gated receptors (e.g. NMDAR) and VGCCs triggers intracellular signalling cascades that remain spatially confined and act locally to regulate cellular processes ^136^. In the soma, the opening of L-type VGCCs causes a rise in intracellular Ca ^2+^ and thereby activates Ca ^2+^-dependent signalling molecules that shuttle from the cytoplasm to the nucleus such as activated ERK and NF-κB ^74^. Moreover, it may also allow the generation of RyR- or IP _3_R-dependent Ca ^2+^ waves that propagate along the ER from the plasma membrane to the nucleus inducing the increase of Ca ^2+^ concentration within the nucleus ^76^. In addition, it is anticipated that Ca ^2+^-transduction pathways crosstalk with simultaneously activated signalling cascades governed by other second messengers such as IP _3_ and cAMP that will promote or restrain the spread of the Ca ^2+^ signal and will have a profound influence on the precise timing and extent of gene induction. Future research will need to determine how these coordinated processes contribute to shape neuronal plasticity.
