# Supplementary material for: Neuronal activity-regulated gene transcription: how are distant synaptic signals conveyed to the nucleus?
Source: F1000Res. 2012 Dec 19;1:69. [Version 1] doi: 10.12688/f1000research.1-69.v1 (PMC3752646; doi:10.12688/f1000research.1-69.v1)
Supplement: Peer review history

# Peer review: Referee response for version 1

**Published:** 2013-01-23

**Authors:** Anne West

**Affiliations:** Department of Neurobiology, Duke University, Durham, NC, USA

This is a concise, well-written review on synapse to nucleus signaling that does an exemplary job not only of reviewing what experiments have been done in this field but also laying out what data are required to more fully address each of the models proposed.

I have read this submission. I believe that I have an appropriate level of expertise to confirm that it is of an acceptable scientific standard.

# Peer review: Referee response for version 1

**Published:** 2013-01-11

**Authors:** Michael Shiflett

**Affiliations:** Department of Psychology, Rutgers University, Newark, NJ, USA

This is a well-written review on the possible mechanisms linking synaptic activity to gene transcription. The author provides a thoughtful consideration of technical and analytic challenges confronted by researchers addressing this question. The outline of a model of how synaptic activity signals are conveyed to the nucleus is given. This model, in conjunction with local synaptic 'tagging' hypotheses, provides a clearer picture of a complex process underlying long-term changes at synapses.

I have read this submission. I believe that I have an appropriate level of expertise to confirm that it is of an acceptable scientific standard.
